# FACTORS RELATED TO QUALITY OF LIFE OF OLDER PEOPLE IN TEHRAN IN 2019

**DOI:** 10.1093/geroni/igz038.3279

**Published:** 2019-11-08

**Authors:** Ali Darvishpoor Kakhki, Zhila Abedsaeedi, Patricia Davidson

**Affiliations:** 1 Shahid Beheshti University of Medical Sciences, Tehran, Iran (islamic Republic Of); 2 Johns Hopkins University, Baltimore, Maryland, United States

## Abstract

Aging is accompanied by a variety of challenges and tensions that have consequences for quality of life in older people. Optimizing quality of life is a key goal for active aging. This study was conducted to describe factors and quality of life of older people in the city of Tehran. This descriptive, correlational study was conducted on a sample of 290 older people above age 60 recruited in 10 public parks in five regions of Tehran in 2019. A socio-demographic questionnaire and instrument on Quality Of Life of Older People (QOL-OP) were used for data collection. Content validity and Cronbach’s alpha were used for evaluation of the validity and reliability of questionnaires. Data were analyzed with SPSS software. Fifty-one percent of older people were male and 49% male with a mean age of 69.52(± 7.11) years. The mean scores for quality of life domains ranged from 57.61(±7.11) for family integrity, to 64.73(±7.50) for spiritual well-being on a 100-point scale. The scores on health-related quality of life domains were influenced significantly by characteristics of age, gender, marital status, housing status, income source, treatment insurance, supplementary treatment insurance, education level, living persons, number of offspring, and annual physician referral rates. The findings of this study showed that quality of life of older people was lower than expected and related to a number of factors. Monitoring modifiable factors such as treatment insurance and non-modifiable factors will help us to preserve or improve quality of life of older people.

